# MicroRNA-146 and cell trauma down-regulate expression of the psoriasis-associated atypical chemokine receptor ACKR2

**DOI:** 10.1074/jbc.M117.809780

**Published:** 2017-12-26

**Authors:** Kave Shams, Mariola Kurowska-Stolarska, Fabian Schütte, A. David Burden, Clive S. McKimmie, Gerard J. Graham

**Affiliations:** From the ‡ Skin Research Group, Leeds Institute of Rheumatic and Musculoskeletal Medicine, National Institute for Health Research Biomedical Research Centre and; ‡‡ Virus Host Interaction Team, Leeds Institute of Cancer and Pathology, University of Leeds, St. James' University Hospital, Leeds LS9 7TF, United Kingdom,; § Department of Dermatology, Chapel Allerton Hospital, Leeds LS7 4SA, United Kingdom,; ¶ Chemokine Research Group and; ‖ Institute of Infection, Immunity and Inflammation, 120 University Place, University of Glasgow, Glasgow G12 8TA, Scotland, United Kingdom, and; ** Department of Dermatology, Lauriston Building, Edinburgh EH3 9HA, Scotland, United Kingdom

**Keywords:** chemokine, immunology, inflammation, microRNA (miRNA), psoriasis

## Abstract

Chemokines are the principal regulators of leukocyte migration and are essential for initiation and maintenance of inflammation. Atypical chemokine receptor 2 (ACKR2) binds and scavenges proinflammatory CC-chemokines, regulates cutaneous T-cell positioning, and limits the spread of inflammation *in vivo*. Altered ACKR2 function has been implicated in several inflammatory disorders, including psoriasis, a common and debilitating T-cell–driven disorder characterized by thick erythematous skin plaques. ACKR2 expression is abnormal in psoriatic skin, with decreased expression correlating with recruitment of T-cells into the epidermis and increased inflammation. However, the molecular mechanisms that govern ACKR2 expression are not known. Here, we identified specific psoriasis-associated microRNAs (miRs) that bind ACKR2, inhibit its expression, and are active in primary cultures of human cutaneous cells. Using both *in silico* and *in vitro* approaches, we show that miR-146b and miR-10b directly bind the ACKR2 3′-UTR and reduce expression of ACKR2 transcripts and protein in keratinocytes and lymphatic endothelial cells, respectively. Moreover, we demonstrate that ACKR2 expression is further down-regulated upon cell trauma, an important trigger for the development of new plaques in many psoriasis patients (the Koebner phenomenon). We found that tensile cell stress leads to rapid ACKR2 down-regulation and concurrent miR-146b up-regulation. Together, we provide, for the first time, evidence for epigenetic regulation of an atypical chemokine receptor. We propose a mechanism by which cell trauma and miRs coordinately exacerbate inflammation via down-regulation of ACKR2 expression and provide a putative mechanistic explanation for the Koebner phenomenon in psoriasis.

## Introduction

Chemokines are members of a large family of chemotactic cytokines that are the primary *in vivo* regulators of leukocyte migration. Chemokines are central to the pathogenesis of inflammatory diseases (1, 2) and interact with leukocytes through members of the seven-transmembrane–spanning family of G-protein–coupled receptors (3) to orchestrate the recruitment of inflammatory cells into, and within, tissues. Chemokines and their receptors are broadly categorized as being either inflammatory or homeostatic according to the *in vivo* contexts in which they function. Importantly, in addition to the classical signaling chemokine receptors, there exists a subfamily of chemokine-binding seven-transmembrane–spanning molecules that are referred to as atypical chemokine receptors (ACKRs).^3^ The ACKRs are promiscuous in their ligand binding, tend to be expressed on stromal cells, and are unable to mediate typical chemokine-induced signaling responses following ligand binding (4–6). We have a particular interest in one of these, ACKR2 (previously known as D6), which is a high-affinity receptor for multiple inflammatory CC-chemokines (5, 7, 8). ACKR2 does not mount classical signaling responses following ligand binding (9) but internalizes ligands and targets them for intracellular degradation (10, 11). Thus, ACKR2 functions as a scavenger of proinflammatory chemokines, and its dysfunction has been implicated in numerous inflammatory diseases (6). In addition, ACKR2 is involved in regulating a range of inflammation-dependent developmental processes (12, 13).

ACKR2 expression is elevated in many human inflammatory conditions, including rheumatoid arthritis (14), systemic sclerosis (15), and psoriasis (16). Psoriasis is a common systemic inflammatory disease with profound effects associated with both excess morbidity and mortality (17, 18). Psoriasis is typically characterized by clearly demarcated thick erythematous skin plaques with white adherent scales surrounded by extensive areas of apparently normal looking (unaffected) skin. Psoriatic plaques tend to preferentially develop in areas undergoing repeated trauma such as the skin on the elbows and knees (19). Additionally, the Koebner phenomenon is frequently reported in patients with psoriasis whereby relatively simple skin trauma of unaffected skin leads to the rapid development of psoriatic plaques in the vicinity of the preceding trauma (20).

In healthy skin, ACKR2 is primarily expressed by dermal lymphatic endothelial cells and keratinocytes. ACKR2 expression in these cells helps to compartmentalize tissue inflammatory responses to insult and infection by controlling the position of inflammatory leukocytes (21–23). We have recently shown that the spread of psoriasiform inflammation to unaffected cutaneous sites is restricted by selective up-regulation of cutaneous ACKR2 in the unaffected epidermis. At these sites, high ACKR2 expression in keratinocytes limits local chemokine activity and suppresses entry of T-cells into the epidermis, thereby protecting against the development of plaques in uninvolved skin. In contrast, skin in which ACKR2 expression is relatively reduced is associated with enhanced inflammatory chemokine activity, increased numbers of infiltrating T-cells in the epidermis, and the emergence of inflammatory plaques (24). The factors that trigger nascent plaque development in psoriasis are not well understood, although our previous data suggest that one such factor includes simple skin trauma, which induces down-regulation of epidermal ACKR2 (16).

Despite the importance of epidermal ACKR2 in regulating psoriasiform inflammation and its transcriptional response to cutaneous trauma, the molecular mechanisms by which ACKR2 expression is regulated in keratinocytes are not understood. Here, by utilizing a combination of *in silico* and *in vitro* approaches, we identify two psoriasis-associated microRNAs that are up-regulated by trauma in primary cultures of human keratinocytes. We show that the identified microRNAs bind the ACKR2 3′-untranslated region (3′-UTR), resulting in decreased expression at the transcript and protein levels. As such, this is the first demonstration of known disease-associated miRNAs regulating atypical chemokine receptor expression and thereby modulating positioning of inflammatory leukocytes within the skin. Importantly, our study highlights a novel molecular mechanism by which trauma leads to the development of new plaques in psoriasis (the Koebner phenomenon).

## Results

### Three psoriasis-associated microRNAs are predicted to bind the ACKR2 3′-UTR

**Figure 1.**
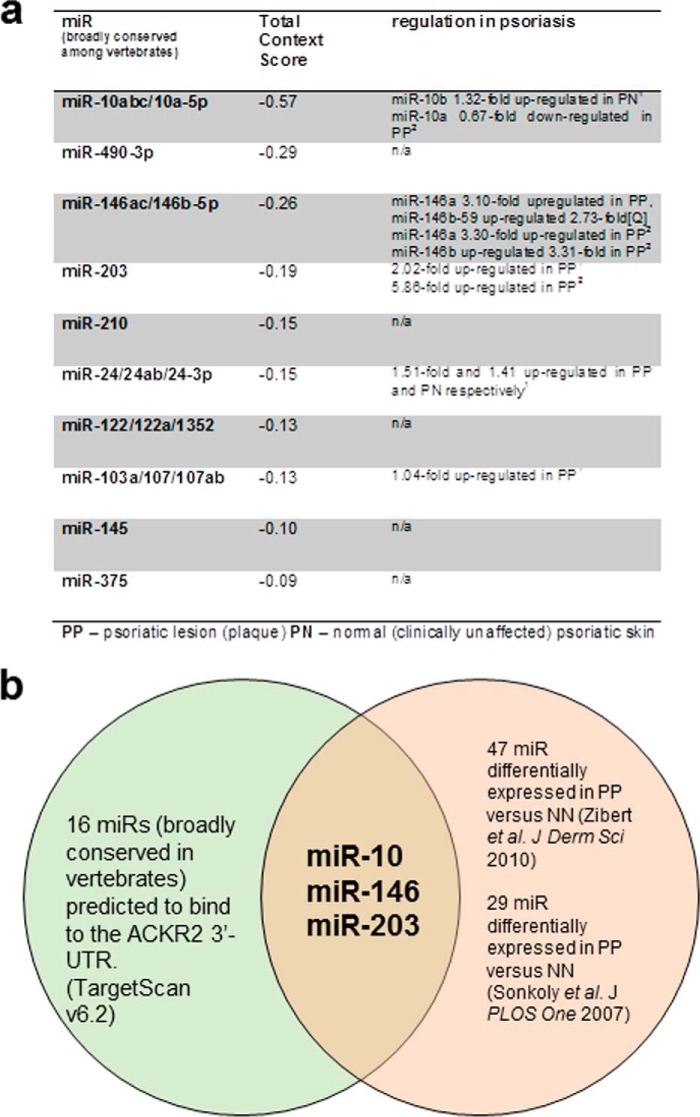
***In silico* analyses identified several psoriasis-associated putative ACKR2 3′-UTR–interacting microRNAs.** *a*, summary of microRNAs predicted to bind the ACKR2 3′-UTR with associated microRNA-specific data from previous psoriasis publications. The 10 microRNAs most likely to target ACKR2 are included in the diagram with relevant findings in two papers given in the *right-hand column*. miR-10, miR-146, and miR-203 are all differentially regulated in psoriasis (27, 37). In the figure, Footnote 1 is Ref. 37, and Footnote 2 is Ref. 27. *b*, three microRNAs (miR-10, miR-146, and miR-203) are predicted to bind the ACKR2 3′-UTR and are differentially expressed in psoriasis. The diagram shows microRNAs that are predicted to bind the ACKR2 3′-UTR *in silico* and that have been shown to be differentially expressed in psoriasis by microarray studies.

MicroRNAs have emerged as the most abundant class of gene regulators and have been implicated in a range of inflammatory disease processes. They predominantly act as negative regulators of gene expression at a post-transcriptional level (25). MicroRNAs bind their mRNA target 3′-UTR, which leads to mRNA degradation. Typically, one microRNA binds multiple mRNA targets, and the same mRNA 3′-UTR can be targeted by numerous microRNAs; this promiscuity introduces a significant degree of complexity in microRNA/target interactions and subsequent regulation of gene expression (26). Recent studies have shown that many microRNAs are differentially expressed in psoriasis with a large number being significantly overexpressed in the psoriatic plaques (27). Given their abundance as negative regulators of gene expression, microRNAs are thus plausible regulators of ACKR2 expression. Accordingly, we used the bioinformatics database TargetScan to identify possible microRNA targets on the ACKR2 3′-UTR. As ACKR2 is present throughout mammals, results were filtered such that only microRNAs that are broadly conserved among vertebrates would be identified to increase the likelihood of identified microRNA species being biologically relevant. Using this search strategy, 16 microRNAs were identified that were both 1) predicted to bind the 3′-UTR of human ACKR2 and 2) broadly conserved among vertebrates (the top 10 hits are shown in Fig. 1*a*). To further focus in on those microRNAs of relevance to psoriasis pathogenesis, we compared the list of microRNAs that are predicted to bind the 3′-UTR of ACKR2 with microRNAs previously shown to be differentially expressed in psoriatic plaques where ACKR2 expression is reduced relative to the surrounding tissue (16). In this way, we identified three microRNAs that were present in both lists, miR-10, miR-146, and miR-203. Notably, each of these miRNAs had a particularly high *in silico* likelihood of regulating ACKR2 (Fig. 1, *a* and *b*). Additionally, these three microRNAs have each been shown to play roles in skin homeostasis and inflammation (28–30). They are thus plausible epigenetic regulators of ACKR2 expression and were selected for further evaluation.

### miR-146b and miR-10b reduce the expression of ACKR2 mRNA in primary human keratinocytes (KCs) and lymphatic endothelial cells (LECs), respectively

**Figure 2.**
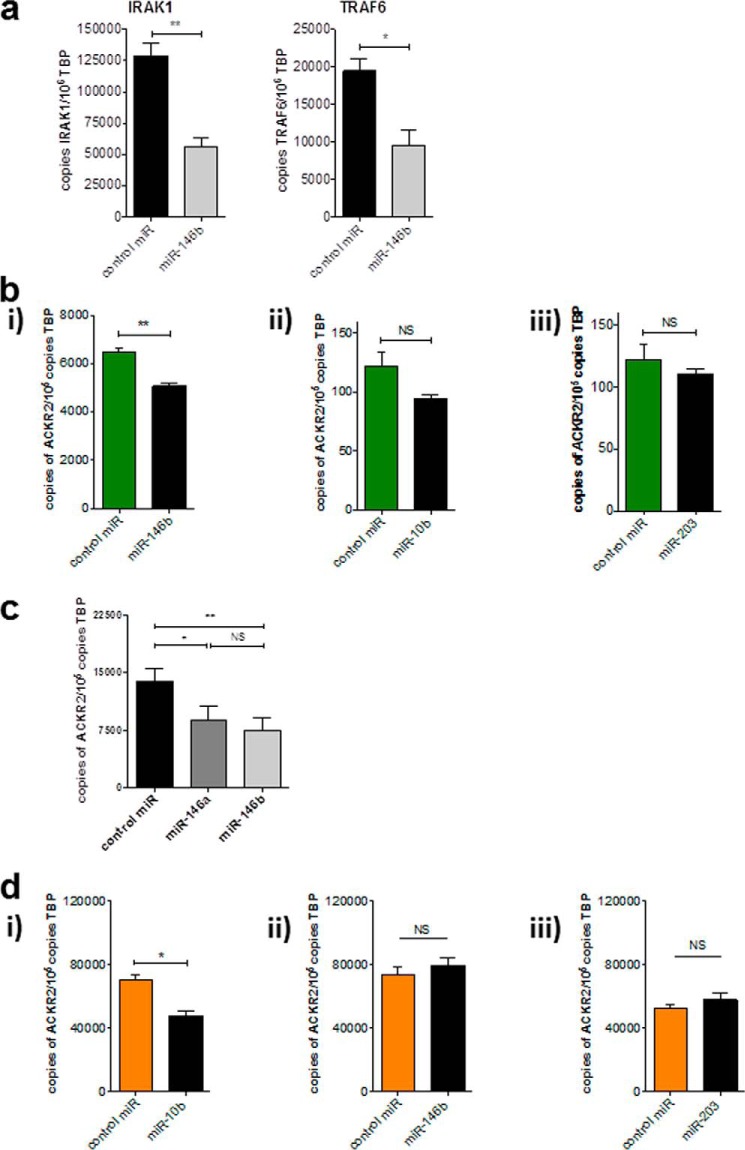
**miR-146 and miR-10 transfection reduced AKCR2 transcripts in KCs and LECs, respectively.** *a*, absolute quantification of IRAK/TRAF6 mRNA (previously validated miR-146b targets) in primary healthy human keratinocytes that were stimulated with 100 ng/ml human recombinant IFNγ prior to transfection. *b*, absolute quantification of ACKR2 transcripts following transfection of KCs with miR-146b (*i*), miR-10 (*ii*), and miR-203 (*iii*). *c*, absolute quantification of ACKR2 transcripts following transfection of KCs with miR-146a and miR-146b. *d*, absolute quantification of ACKR2 transcripts following transfection of LECs with miR-10 (*i*), miR-146b (*ii*), and miR-203 (*iii*). In all cases, cells were transfected for 24 h and left for a further 24 h prior to lysis and RNA extraction. microRNAs were added at 10 nm; control is scrambled microRNA. Shown are representative experiments conducted in cells from various cell donors. Significance was assessed using Student's *t* test (*, *p* < 0.05; **, *p* < 0.01) except for *d* where significance was assessed using one-way ANOVA with Tukey's post-test (*, *p* < 0.05; **, *p* < 0.01). *Error bars* represent S.E. *NS*, not significant; *TBP*, TATA-binding protein.

KCs and LECs are the main ACKR2-expressing cells in skin (16, 31). To ensure that microRNAs could be successfully transfected into primary KCs, cells were transfected with miR-146b (most strongly up-regulated miRNA in psoriatic plaques; Fig. 1*A*) for 24 h, and the expression of two IFNγ-induced genes known to be down-regulated by miR-146b (IRAK1 and TRAF6) was determined by Q-PCR (Fig. 2*a*). The data show a clear ability of miR-146b to down-regulate levels of both transcripts, thus demonstrating that microRNA could be successfully delivered into the cytoplasm of KCs. Primary KCs from at least two separate healthy donors were used for all subsequent experiments. Transfection of KCs with miR-146b significantly suppressed ACKR2 mRNA expression; however, miR-10b and miR-203 had no significant effects on ACKR2 transcript levels (Fig. 2*b*).

miR-146a and -b are differentially expressed in psoriasis, suggesting non-redundant roles in this context (27), although our *in silico* analyses predicted that miR-146a and miR-146b both bind the ACKR2 3′-UTR (Fig. 1*a*). To determine whether any differences exist between miR-146a and miR-146b in the regulation of ACKR2 expression, the two were directly compared (Fig. 2*c*). Both miR-146a and miR-146b mediated a similar reduction in ACKR2 mRNA expression in KCs after 24 h with no significant differences between the two variants. Thus, both miR-146a and miR-146b down-regulate ACKR2 transcript levels in KCs. Next the three microRNAs were transfected into primary healthy human dermal LECs, which also express high levels of ACKR2. Only miR-10b significantly reduced ACKR2 mRNA expression in LECs (Fig. 2*d*). Thus, these data demonstrate that psoriasis-associated microRNAs are capable of regulating ACKR2 expression in keratinocytes and lymphatic endothelial cells.

### miR-146b/miR-10b bind directly to the ACKR2 3′-UTR

**Figure 3.**
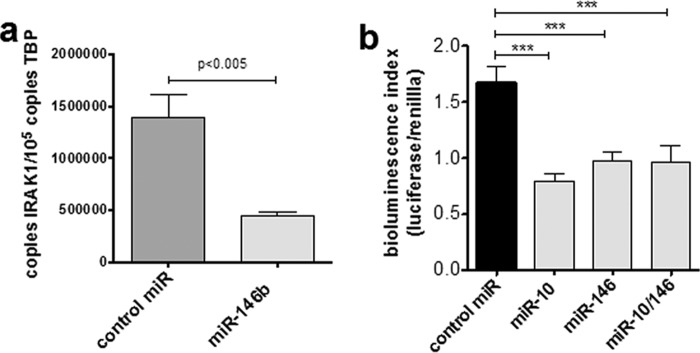
**miR-146b and miR-10b functionally interact with the ACKR2 3′-UTR.** *a*, miR-146b can be successfully transfected into HEK293 cells. Absolute quantification of IRAK1 mRNA was assessed by Q-PCR in HEK293 cells following transfection with miR-146b as compared with scrambled control. *b*, ACKR2 3′-UTR was cloned into the downstream UTR of a firefly luciferase reporter, and interaction with transfected miRs was determined (bioluminescence inversely proportional to microRNA 3′-UTR binding) and normalized to *Renilla* luciferase. Results are representative from two different luciferase-expressing HEK293 clones following transfection with miR-10b and miR-146b (singly and in combination) and scrambled miR control. Significance was assessed using one-way ANOVA (***, *p* < 0.005). *Error bars* represent S.E. *TBP*, TATA-binding protein.

To determine whether miR-146b and miR-10b reduce ACKR2 transcripts through direct interaction with the ACKR2 3′-UTR, the 3′-UTR of ACKR2 was cloned into a Dual-Luciferase reporter vector (pmiRGLO vector). The ACKR2 3′-UTR containing putative miRNA target sites was inserted immediately 3′ of a phosphoglycerate kinase promoter-driven firefly luciferase gene to evaluate ACKR2 3′-UTR–dependent microRNA interactions on transcription. This construct was transfected into HEK293 cells, and stable clones were selected. These clones were then confirmed as being amenable to microRNA transfection by assessing IRAK1 expression, which was significantly reduced following miR146b transfection (Fig. 3*a*). Having demonstrated that HEK cells could be transfected with functional microRNAs of interest, we next determined whether miR-10 and miR-146b transfection modulated luciferase activity (Fig. 3*b*). Both miR-10 and miR-146b transfection significantly down-regulated luciferase activity in HEK cells, although dual transfection with both miRs did not result in an additive decrease. Thus, both miR-10 and miR-146b can mediate a decrease in transcript levels through direct interactions with the ACKR2 3′-UTR.

### miR-146b transfection of KC and miR-10b transfection of LEC reduced ACKR2 protein expression

**Figure 4.**
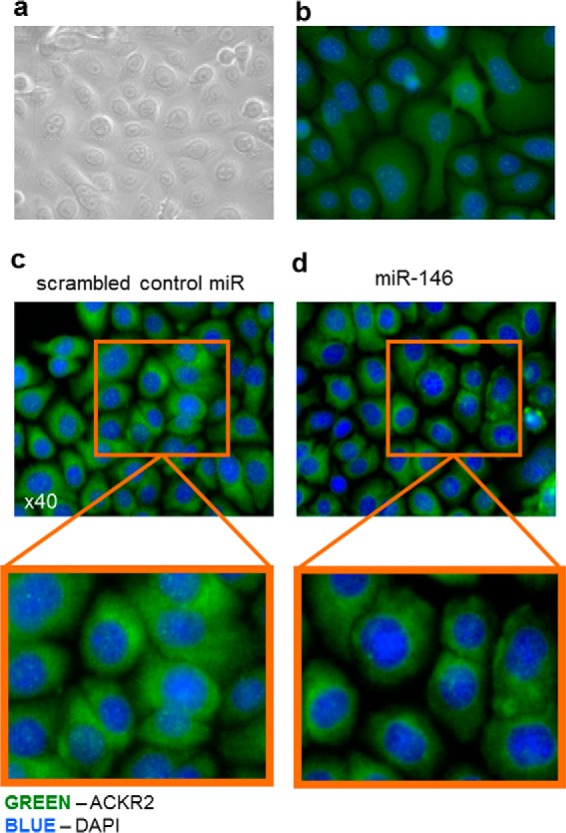
**Transfection of KCs with miR-146b reduced cytoplasmic ACKR2 protein distribution.** *a*, representative bright-field image of KCs grown as a confluent monolayer. *b*, representative immunofluorescence of KCs grown as confluent monolayers and stained with isotype control antibody. *c* and *d*, representative immunofluorescence microscopy images of confluent monolayers of KCs 48 h after transfection with scrambled miR control (*c*) or miR-146b (*d*).

Next we determined the effect of miR-146b on ACKR2 protein expression and distribution in primary KCs and LECs. For reasons that are not immediately obvious, we have been unable to successfully apply our functional ACKR2 assay (31) to primary keratinocytes. We have therefore relied on immunofluorescence analysis to measure ACKR2 protein levels. Fully confluent human KC monolayers (Fig. 4*a* with isotype control shown in Fig. 4*b*) were transfected with miR-146b for 48 h, and ACKR2 protein expression was determined by immunocytochemistry. Bright green punctate cytoplasmic staining, typical of ACKR2 expression in other cell types (11, 32), was observed in KCs transfected with scrambled miR control (Fig. 4*c*). This staining was more marked in the perinuclear region and often in an asymmetrical fashion in keeping with higher ACKR2 levels in the endoplasmic reticulum. This immunofluorescence staining pattern is as expected, and we have previously published that the majority of ACKR2 protein is found within intracellular vesicles that traffic to, and from, the cell surface (11, 32). In contrast, when monolayers of KCs were transfected with miR-146b, cells exhibited a loss of ACKR2 staining throughout the cytoplasm, although the perinuclear staining was still evident (Fig. 4*d*).

**Figure 5.**
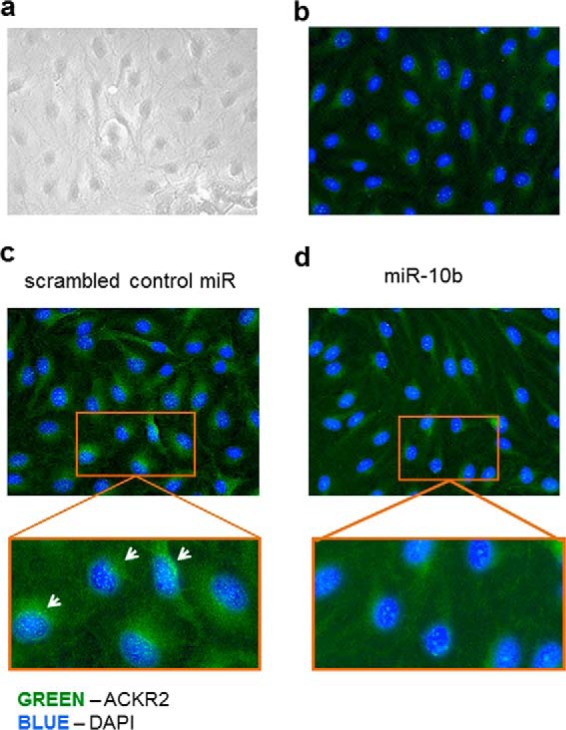
**Transfection of LECs with miR-10 reduced ACKR2 protein expression throughout the cytoplasm.** *a*, representative bright-field image of LECs grown as a confluent monolayer. *b*, representative immunofluorescence of LECs grown as confluent monolayers and stained with isotype control antibody. *c* and *d*, representative immunofluorescence microscopy images of confluent monolayers of LECs 48 h after transfection with scrambled miR control (*c*) or miR-10 (*d*). *White arrows* indicate asymmetric distribution of ACKR2.

To investigate the effect of miR-10b on ACKR2 expression in human LECs, LECs that had been grown as confluent monolayers were transfected with miR-10b, and ACKR2 protein expression was determined by immunofluorescence after 48 h (Fig. 5*a* with isotype control shown in Fig. 5*b*). In keeping with the known difference in ACKR2 transcript level, the level of ACKR2 protein staining was less intense in LECs compared with KCs. The granular staining in LECs was again more marked to one side of the perinuclear region (akin to what was observed in KCs), becoming especially apparent at higher magnification (Fig. 5*c*). Importantly, ACKR2 staining was more pronounced and more granular in scrambled miR control–transfected LECs compared with miR-10b–transfected LECs in which staining was not higher than background autofluorescence (Fig. 5, *b* and *d*). Taken together, immunofluorescence staining of cultured cell monolayers demonstrated that transfection with miR-146b in KCs and miR-10b in LECs led to a reduction in ACKR2 staining in both cell types by 48 h.

### Tensile cell trauma of cytokine-treated KC leads to a rapid reduction in ACKR2 mRNA expression

Next we examined the possible relevance of our finding for cutaneous inflammatory responses such as those typical of psoriasis. Psoriatic patients have elevated cutaneous ACKR2 expression in unaffected skin, which mouse models suggest may offer protection from further lesion development (24). Superficial trauma of unaffected skin in psoriatic patients can trigger psoriatic plaque development (koebnerization) concurrent with ACKR2 expression down-regulation (16, 33, 34). This phenomenon is a feature of many psoriatic patients where lesions have a particular predilection for sites that undergo repeated stretching in daily life (*e.g.* knees, elbows, and skin folds) (20). Thus, we wanted to determine whether tensile trauma down-regulated ACKR2 in cultured keratinocytes in a cell-autonomous manner and whether this correlated with increased miR-146 activity. To specifically determine the effect of tensile cell trauma on ACKR2 expression, a reductionist *in vitro* model was developed. The model developed was based on the FlexCell^TM^ International FX5000 machine whereby primary human cells were grown on silicone membranes. The membrane was subsequently subjected to repeated biaxial cyclical tension *in vitro* with a predetermined degree of tension, stretch waveform, and cycle number (this model is summarized in Fig. S1). Neither KCs nor LECs adhered to uncoated silicone, and therefore KCs were grown on collagen I–coated silicone, whereas LECs were grown on fibronectin-coated silicone. This was found to be associated with stable cell attachment over a 12-h stretch cycle for each cell type.

**Figure 6.**
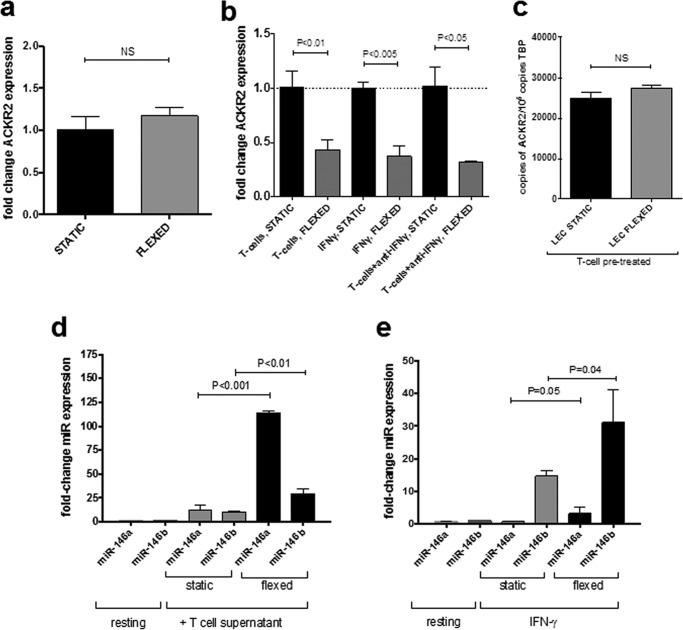
**Effect of tensile stress on ACKR2 expression by primary human KCs.** *a–c*, absolute quantification of ACKR2 mRNA normalized to TATA-binding protein (*TBP*) in keratinocytes. *a*, healthy human primary KCs that remained static or were subjected to tensile stress (flexed) at 0.8 Hz for 12 h and then allowed to rest for 12 h prior to cell lysis and RNA extraction. *b*, healthy human primary KCs that remained static or were subjected to tensile stress (flexed) at 0.8 Hz for 12 h and then allowed to rest for 12 h prior to cell lysis and RNA extraction. KCs were pretreated with either 1) tissue culture supernatant from activated human T-cells (1:8 dilution in fresh medium), 2) 100 ng/ml recombinant human IFNγ, or 3) tissue culture supernatant from activated human T-cells plus neutralizing anti-IFNγ antibodies overnight prior to flexing at 0.8 Hz for 12 h. *Black bars*, non-flexed static controls; *gray bars*, flexed cells. Significance was assessed using one-way ANOVA. *c*, treatment of healthy primary LECs overnight with tissue culture supernatant from activated human T-cells (diluted 1:8 with fresh medium) prior to flexing. *d* and *e*, effect of tensile stress on miR-146 expression in inflamed keratinocytes. -Fold change in miR-146a and miR-146b expression was assessed by Q-PCR and normalized to scrambled miR–treated static KCs. KCs were treated for 16 h with either tissue culture supernatant from activated human T-cells (1:8 dilution in fresh medium) (*d*) or recombinant IFN-γ at 100 ng/ml (*e*) prior to flexing at 0.8 Hz for 12 h. *Error bars* represent S.E. *NS*, not significant.

To examine the effect of tensile trauma on KC ACKR2 expression, confluent KC monolayers were subjected to stretching for 12 h during which the membrane was stretched by 15% every 0.8 s (0.8 Hz), which is not atypical for skin sites such as elbows that are exposed to daily repeated stretching. Cells were rested for a further 12 h before lysis to enable gene expression and ACKR2 expression determined by Q-PCR. The data obtained (Fig. 6*a*) showed that stretching of non-inflamed KCs did not result in altered ACKR2 expression. However, these primary human cells are derived from healthy donors and are grown in the absence of typical psoriasis-associated factors. Psoriasis patients have markedly elevated T-cell cytokines, including IFNγ, that correlate with elevated ACKR2 in unaffected skin (16, 24). Therefore, to better model human psoriasis where KCs exist in the context of systemically elevated T-cell cytokines, KCs were treated with either tissue culture supernatant from activated human T-cells or recombinant IFNγ (both of which up-regulate ACKR2 expression (16, 24)) and then exposed to tensile stress for 12 h at 0.8 Hz. In contrast to non-treated KCs (Fig. 6*a*), tensile trauma of KCs pretreated with T-cell supernatants led to a significant decrease in ACKR2 expression (Fig. 6*b*). This was also the case for IFNγ-pretreated KCs, suggesting that KCs with elevated ACKR2 levels such as those found in psoriasis patients display tensile stress–induced down-regulation of expression. Interestingly, treatment of the T-cell supernatant with IFNγ-neutralizing antibodies did not diminish this effect, suggesting that soluble T-cell products other than IFNγ can mediate the observed effect (Fig. 6*b*). In contrast to KCs, there was no effect on ACKR2 expression upon flexing of LECs that were stimulated with T-cell supernatant (Fig. 6*c*). Thus, the response of inflamed KC and LECs to tensile stress appears to be different even when cells have been similarly pretreated.

To determine whether there was a possible link between these observations and alterations in miR-146b expression, miR-146a/b expression levels were quantified by Q-PCR in inflamed KCs. Both miR-146a and miR-146b were up-regulated in KCs that had been treated with tissue culture supernatant from activated human T-cells compared with resting non-inflamed KCs (Fig. 6*d*, *gray bars*). Importantly, when these cells were subjected to tensile trauma, this effect was dramatically amplified; there was an over 100-fold induction of miR-146a and a lesser (albeit significant) induction of miR-146b (Fig. 6*d*, *black bars*). A similar observation was found for KCs pretreated with recombinant IFNγ and then subjected to stretching, although in this case miR-146b was more strongly induced than miR-146a (Fig. 6*e*). Taken together, these data suggest that KCs exposed to psoriasis-associated T-cell cytokines markedly up-regulate miR-146a/b expression concurrently with ACKR2 down-regulation upon stretching and provide a putative mechanistic link that explains the Koebner phenomenon in psoriasis.

## Discussion

The atypical chemokine receptor ACKR2 is expressed in barrier tissues, including the skin, and functions as a high-capacity scavenger of proinflammatory CC-chemokines. *In vivo* and specifically in the context of psoriasiform inflammation, we have shown that ACKR2 restricts T-cell entry into the epidermis and limits inflammation. Additionally, ACKR2 expression is deficient in psoriatic plaques in humans, which may enable uncontrolled inflammation and thereby plaque formation. The mechanism by which ACKR2 down-regulation occurs has, until now, not been described. Indeed, although some proinflammatory cytokines are known to increase expression of ACKR2 (although notably not the psoriasis-associated cytokine IL-17 (16, 24)), little is known about the molecular mechanisms that control atypical chemokine receptor expression. Here, we identify microRNAs that are both differentially expressed in psoriasis and predicted to target the ACKR2 3′-UTR. Experimental evidence is provided that miR-146 and miR-10 bind directly to the ACKR2 3′-UTR and lead to a down-regulation of ACKR2 at transcript and protein levels in KCs and LECs, respectively.

It is notable that we saw, at best, a 50% reduction in luciferase activity, or ACKR2 transcript levels, in cells transfected with miR-146 species. It is widely accepted that this level of transcript knockdown represents a typical response to miR-mediated suppression and that more extensive depletion of target mRNA levels is not commonly seen (35, 36).

We have previously shown that mild trauma (tape-stripping, which induces a mild degree of epidermal damage and tensile stress) to uninvolved psoriatic skin leads to rapid transcriptional down-regulation of ACKR2, which, we propose, allows chemokine-dependent inflammation to become established and contributes to the Koebner phenomenon and development of plaques (16). The Koebner phenomenon is reported in psoriasis as well as a range of other skin diseases whereby skin trauma (for example scratching and tape-stripping) triggers the appearance of the underlying disease at those sites (20). However, tape-stripping induces several types of damage to skin, including disruption of skin barrier function as well as tensile stress during the rapid removal of the tape adherent to skin. Additionally, it is notable that psoriatic plaques have a predilection for areas undergoing repeated tension, *e.g.* elbows, knees, and skin folds. To specifically mimic tensile trauma to keratinocytes *in vitro*, we used a cell-flexing device to induce tensile stretch across the length of the keratinocytes. Flexing of resting keratinocytes, which express very low ACKR2 levels, did not alter transcript levels. In comparison, flexing of keratinocytes pretreated with conditioned medium from activated human T-cells significantly reduced elevated ACKR2 expression. This was also seen with stretching of IFNγ-treated keratinocytes. Together, our results demonstrate that, although keratinocyte trauma does not alter ACKR2 expression under “resting” conditions, it does nonetheless significantly reduce T-cell–induced ACKR2 expression levels, thus mimicking what is seen in uninvolved human psoriatic skin upon trauma (16). Despite being well described, a mechanism for the Koebner phenomenon (koebnerization) has not been elucidated. Our findings presented here provide a novel molecular mechanism by which koebnerization might be induced in unaffected psoriatic skin, potentially through the up-regulation of miR-146 and its interaction with ACKR2 transcripts. To this end, we defined the impact of flexing on miR-146 expression in T-cell supernatant–treated keratinocytes. These analyses revealed a marked induction of miR-146a (and to a lesser extent miR-146b) in flexed, compared with static, T-cell–treated keratinocytes. These data therefore indicate that increased expression of miR-146 following mild trauma to keratinocytes, and in psoriatic plaques, is able to counteract the inductive effects of activated T-cell products on ACKR2 expression. Thus, these findings also provide a plausible mechanistic explanation for the Koebner phenomenon in inflammatory skin diseases such as psoriasis and may have wider implications for non-cutaneous inflammatory diseases in which tensile strength is a contributing factor, *e.g.* rheumatoid arthritis.

## Experimental procedures

### MicroRNA in silico selection

MicroRNAs that were predicted to bind to the 3′-UTR of ACKR2 were identified using TargetScan version 6.2 (www.targetscan.org).^4^ MicroRNAs with the highest likelihood of binding the ACKR2 3′-UTR (as indicated by the total context, *C_T_*, score) and that were concurrently identified as being up-regulated in psoriatic plaques in humans were selected for further study (27, 37).

### Cell culture

Primary healthy human KCs and LECs were purchased (PromoCell, Heidelberg, Germany) and cultured according to the manufacturer's instructions in KCGM2 and ECMV2 medium, respectively (PromoCell). All *in vitro* experimental work was conducted at passage 3–4 at 70% confluence (averaged across the vessel). All cells were maintained at 37 °C in a humidified tissue culture incubator with 5% CO_2_. Cells were grown in the presence of 1% penicillin/streptomycin (Sigma) and 0.1% gentamicin solution (50 mg/ml; Sigma). Experiments in primary human cells were generally repeated using cells from at least two separate cell donors. HEK293 cell lines were kindly donated by Dr. K. Hewit and grown in DMEM (Sigma) to which was added 50 ml of fetal calf serum and 5 ml of l-glutamine/500 ml (Sigma). Data representative of two or more independent experiments with a minimum of three replicates for each experiment.

### MicroRNA transfection of cells

Cells were transfected with Lipofectamine RNAiMAX according to the manufacturer's instructions (Thermo Fisher). Cell culture medium was changed to antibiotic-free equivalents 24 h prior to transfection and maintained antibiotic-free throughout the transfection process. Cells were transfected at 70% confluence. MicroRNAs, scrambled microRNA control, and miR inhibitors (mirVana, Thermo Fisher) were transfected according to the manufacturer's in 50 μl of Opti-MEM (reduced serum medium; Thermo Fisher). Transfection was allowed to occur at 37° for 24 h before the cells were lysed (or the medium was changed for prolonged incubation). At this concentration, the microRNAs had no detectable effect on cell viability.

### RNA extractions and quantitative PCR

RNA was extracted and purified on RNeasy microcolumns or miRNeasy columns (Qiagen) with on-column DNase (Qiagen) digestion according to the manufacturer's instructions. Whole tissue samples were homogenized in Qiazol with stainless steel beads using a TissueLyser LT (Qiagen). 1 μg of total RNA was reverse transcribed using nanoScript RT or RT^2^ kits according to the manufacturer's instructions (PrimerDesign, UK). For samples intended for microRNA Q-PCR, 440 ng of total RNA was reverse transcribed using an miScript II RT kit according to the manufacturer's instructions (Qiagen). Samples were diluted 1:5 in nuclease-free water prior to being used as a template for Q-PCR. Gene transcripts were quantified by quantitative PCR analysis using Perfecta SYBR Green Master Mix according to the manufacturer's instructions (Quanta, UK). Samples were analyzed in quadruplicate on a 384-well Applied Biosystems 7900HT platform (Life Technologies). ACKR2 transcript levels were normalized to human TATA-binding protein. The sequences for the Q-PCR primers were as reported previously (16, 31, 38) except for TRAF6 and IRAK1. For samples intended for microRNA quantification, Q-PCR was performed using the miScript SYBR Green PCR kit with primers for miR-146a and 146b normalized to RNU6B expression (all from Qiagen and according to the manufacturer's instructions) and analyzed on a 384-well Applied Biosystems 7900HT. Results for microRNA were analyzed according to the ΔΔ method rather than semiabsolute methods as used for mRNA.

### Luciferase microRNA assay

The 3′-UTR of ACKR2 was cloned into the Dual-Luciferase system containing the pmiRGLO vector (Promega). The 3′-UTR region was sequenced to ensure putative microRNA-binding sites were intact (Eurofins, UK).

The plasmid was transfected into HEK293 cells, and stable transfectants were generated through selection with 0.8 mg/ml G418 (Promega). Non-ACKR2–containing pmiR vector was transfected as a control. HEK293 cells were donated by Dr. K Hewit and grown in DMEM (Sigma) with added penicillin/streptomycin as described above. Several clones were identified for further testing, and two of these clones were transfected with the relevant microRNAs/controls, cells were lysed, and firefly luciferase activity was normalized to *Renilla* luciferase (Promega) according to the manufacturer's instructions. pmiR-containing HEK293 cells (*i.e.* with no ACKR2 3′-UTR) and native HEK293 cells were used as controls in the assay, which was repeated using two separate HEK293 clones.

### Fluorescent immunocytochemistry

Cells were cultured in 4-well chamber slides (Thermo Scientific Nunc or BD Falcon). Slides were washed in phosphate-buffered saline (PBS) without calcium (Sigma) and fixed with 100% methanol. Slides were washed and blocked with 20% normal horse serum in PBS with 0.05% Tween (Sigma) followed by an avidin/biotin block. Slides were stained for ACKR2 using Sigma Prestige anti-human ACKR2 IgG antibody (Sigma) raised in rabbit in 2.5% normal human serum and 2.5% horse serum (Vector Laboratories, UK) in PBS-Tween overnight at 4 °C and then stained with a secondary antibody (biotinylated anti-rabbit IgG raised in goat with 5% human serum). Slides were washed, incubated with Avidin-D fluorochrome conjugated with FITC (Vector Laboratories), and mounted with Vectamount containing DAPI (Vector Laboratories) prior to visualization through confocal microscopy (LSM510, Zeiss).

### Tensile stress of keratinocytes

For FlexCell experiments, primary human keratinocytes were grown in BioFlex 6-well plates precoated with collagen I (FlexCell International) for KCs or plain BioFlex 6-well plates coated with fibronectin overnight prior to use (Sigma) for LECs. Cells were subjected to tensile stress at 15% effective stretch, 0.8 Hz, and half-sine waveform using the FlexCell FX5000 cell tension system (FlexCell International). Cells were stretched for 12 h and rested for a further 12 h prior to lysis. All cells were grown and subjected to tension in a humidified incubator at 37 °C with 5% CO_2_.

### T-cell isolation and stimulation

Human T-cells were grown from CD14-depleted human buffy coats from healthy donors and stimulated with concanavalin A (5 ng/ml; Sigma) and grown in RPMI 1640 medium (Life Technologies) with 5% human AB serum and gentamicin (Sigma) in the presence of IL-2 (20 units/ml; Peprotech, UK) from day 4. Cells were purified on Ficoll-Paque after 8 days (GE Healthcare) and grown in the presence of IL-2 alone for 4 days before being activated using CD2/CD3/CD28 beads according to the manufacturer's instructions at a 1:2 bead:cell ratio (Miltenyi Biotec, UK) for 24 h prior to the activated supernatant being removed for downstream applications. Where T-cell supernatant was to be used for KC/LEC stimulation, T-cells were grown in serum-free keratinocyte medium KCGM2 or ECMV2 with added supplements during the 24-h activation period (PromoCell). T-cells used for migration assays were similarly activated albeit at a 1:4 bead:cell ratio for 48 h prior to use.

### Statistical analyses

Student's *t* test, one-way ANOVA, two-way ANOVA, and correlation tests were performed in Prism version 7.0 (GraphPad Software Inc.) with multiple comparison tests as appropriate. *p* < 0.05 was deemed significant. All data are *n* ≥ 3, representative of at least two independent experiments, and given as mean ± S.E. unless otherwise stated.

## Author contributions

K. S., C. S. M., A. D. B., and G. J. G. conceived the study. K. S. and F. S. carried out experimental work. M. K.-S. provided essential advice, insights, and reagents central to the pursuit of the project. All authors were involved in writing the manuscript and all approved the final version for submission.

## Supplementary Material

Supporting Information

## Footnotes

4 Please note that the JBC is not responsible for the long-term archiving and maintenance of this site or any other third party-hosted site.

## Footnotes

3 The abbreviations used are:

ACKR: atypical chemokine receptor
miRNA: microRNA
miR: microRNA
KC: keratinocyte
LEC: lymphatic endothelial cell
Q-PCR: quantitative PCR
ANOVA: analysis of variance.
