# Supplementary material for: MicroRNA-146 and cell trauma down-regulate expression of the psoriasis-associated atypical chemokine receptor ACKR2
Source: J Biol Chem. 2017 Dec 26;293(8):3003–12. doi: 10.1074/jbc.M117.809780 (PMC5827444; doi:10.1074/jbc.M117.809780)
Supplement: Supporting Information [file supp_293_8_3003__index.html]

MicroRNA-146 and cell trauma downregulate expression of the psoriasis-associated atypical chemokine receptor ACKR2 — MicroRNA-146 and cell trauma down-regulate expression of the psoriasis-associated atypical chemokine receptor ACKR2 — MicroRNA regulation of ACKR2 expression — Supporting Information 

# MicroRNA-146 and cell trauma down-regulate expression of the psoriasis-associated atypical chemokine receptor ACKR2

## Supporting Information

- Supplemental data (.docx, 108 KB) - Supplemental data
