# Supplementary material for: MicroRNA-146 and cell trauma down-regulate expression of the psoriasis-associated atypical chemokine receptor ACKR2
Source: J Biol Chem. 2017 Dec 26;293(8):3003–12. doi: 10.1074/jbc.M117.809780 (PMC5827444; doi:10.1074/jbc.M117.809780)
Supplement: Supporting Information [file 10.1074_M117.809780_jbc.M117.809780-1.docx]

**Supplemental Data**

**Shams et al, The psoriasis associated Atypical Chemokine Receptor ACKR2 is regulated by microRNA-146 and cell trauma.**

**Figure S1. Schematic diagram of the cell flexing system used in this study.**

Schematic representation of the cell tension/flexion system.

1. Cells were grown as confluent monolayers on coated silicone membranes (Collagen 1 for KC, Fibronectin for LEC)
2. Cyclical tension was applied to the silicone membrane by means of adjustable vacuum (12h at 0.8Hz)
3. Top view of individual chamber (6-well format) upon application of tensile stress.
